# Individualizing kinase-targeted cancer therapy: the paradigm of chronic myeloid leukemia

**DOI:** 10.1186/s13059-014-0461-8

**Published:** 2014-09-17

**Authors:** Anna M Eiring, Michael W Deininger

**Affiliations:** Huntsman Cancer Institute, The University of Utah, Circle of Hope, Salt Lake City, UT 84112-5550 USA; Division of Hematology and Hematologic Malignancies, The University of Utah, Salt Lake City, UT 84132 USA

## Abstract

The success of tyrosine kinase inhibitors in treating chronic myeloid leukemia highlights the potential of targeting oncogenic kinases with small molecules. By using drug activity profiles and individual patient genotypes, one can guide personalized therapy selection for patients with resistance.

## Introduction

**Figure 1:**
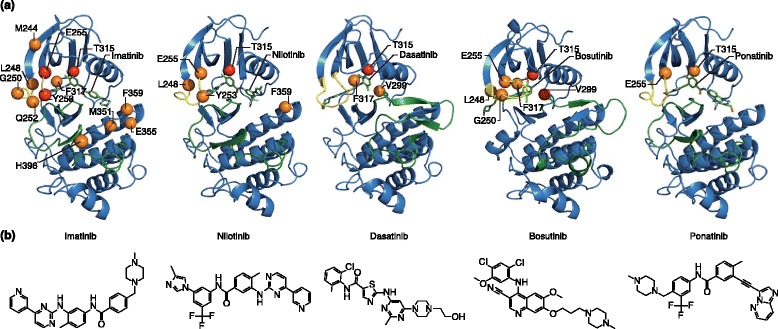
**Tyrosine kinase inhibitors (TKIs) approved for the treatment of chronic myeloid leukemia. (a)** The crystal structure of the ABL1 kinase domain is shown in complex with the indicated TKI. Highlighted residues indicate mutations that confer resistance to the indicated TKI *in vitro*. Orange (moderate) and red (severe) spheres indicate the level of TKI resistance. **(b)** The chemical structures of the TKIs. Adapted with permission from O’Hare *et al*. [3].

Small molecules that inhibit oncogenic signaling pathways are redefining cancer therapy. Potential therapeutic targets have been identified in all physiological processes, reflecting the diversity of mechanisms that promote malignant transformation. In particular, tyrosine and serine/threonine kinases have attracted much attention, which is not surprising given their fundamental role in regulating eukaryotic cellular signaling [1]. Activating mutations in tyrosine and serine/threonine kinases have been identified in many types of cancer and associated with the malignant phenotype, providing a strong therapeutic rationale for the development of small molecule inhibitors that block their activity [2]. The biggest clinical successes to date are the BCR-ABL1 tyrosine kinase inhibitor (TKI) imatinib and its successor compounds, dasatinib, nilotinib, bosutinib and ponatinib (Figure 1). These drugs have transformed chronic-phase chronic myeloid leukemia (CML-CP) from a lethal cancer into a chronic disorder that is compatible with a largely normal span and quality of life.

Chronic myeloid leukemia (CML) is caused by the chimeric tyrosine kinase BCR-ABL1, which results from the t(9;22)(q34;q11) chromosomal translocation and is visible cytogenetically as the Philadelphia chromosome [3]. Resistance to imatinib is frequently caused by mutations in the tyrosine kinase domain of BCR-ABL1, and because the approved TKIs differ in their activity against specific mutants, the clinical selection of TKIs can be driven by *BCR-ABL1* genotype, providing a prime example of personalized therapy in oncology.

Here, we discuss TKI therapy for CML to illustrate the challenges of molecularly targeted cancer therapy, focusing on therapy individualization, the role of clonal evolution and complexity in therapy response and resistance, and how the lessons learned from CML may be applied to TKI therapy in other types of cancer.

## Development of BCR-ABL1 TKIs for CML

Most patients are diagnosed in CML-CP, during which the myeloid cell compartment is expanded but cellular differentiation is maintained [4]. Without effective therapy, CML-CP inexorably progresses to blast phase CML (CML-BP), a disease that resembles an acute leukemia, with complete block of terminal differentiation and a poor prognosis. Murine models indicate that BCR-ABL1 is required and sufficient to induce CML-CP, whereas diverse additional mutations have been implicated in progression to CML-BP (Table 1) [3,5–16].

**Table 1:** **Mutations associated with CML-BP**

**Mutation**	**Percentage prevalence**	**Reference**
Double Ph chromosome	38%	[6]
Isochromosome 17q	30% (myeloid)	[7]
Trisomy 8	53% (myeloid)	[7]
Trisomy 19	23% (myleoid)	[7]
p53 mutations	20-30% (myeloid)	[8]
p16 mutations	50% (lymphoid)	[9]
NUP98-HOXA9 translocations	NR	[10]
AML-EVI1 translocations	NR	[11]
GATA-2 mutations	18% (lymphoid)	[12]
RUNX1 mutations	38% (myeloid)	[13]
CDKN2A/B mutations	50% (lymphoid)	[14]
IKZF1 mutations	55% (lymphoid)	[14]
ASXL1 mutations	20.5% (myeloid)	[16]
TET2 mutations	7.7% (myeloid)	[16]
WT1 mutations	15.4% (myeloid)	[16]
NRAS/KRAS mutations	5.1/ 5.1% (myeloid)	[16]

Ph, Philadelphia; NUP98, nucleoporin 98 kDa; HOXA9, homeobox A9; AML, acute myeloid leukemia; EVI1, ecotropic viral integration site 1; GATA-2, GATA binding protein 2; RUNX1, runt-related transcription factor 1; CDKN2A/B, cyclin-dependent kinase inhibitor 2A/B; IKZF1, IKAROS family zinc finger 1; ASXL1, additional sex combs like transcription regulator 1; TET2, tet methylcytosine dioxygenase 2; WT1, wilms tumor 1; NRAS, neuroblastoma RAS viral oncogene homolog; KRAS, Kirsten rat sarcoma viral oncogene homolog; NR, not reported.

Clinical trials with the first BCR-ABL1 inhibitor, imatinib, were initiated in 1998. The striking activity of imatinib led to rapid regulatory approval for the treatment of patients with CML for whom interferon-α therapy had failed (in 2001), and subsequently to approval for the treatment of newly diagnosed patients (in 2003). Patients with CML-CP who begin treatment with imatinib at diagnosis have an 8-year overall survival of approximately 85%, with an acceptable quality of life [17,18]. Nevertheless, imatinib has limitations: imatinib treatment fails for some 25 to 30% of CML-CP patients because of primary or acquired resistance, and for additional patients due to intolerance [19].

To overcome resistance to imatinib, three second-generation inhibitors have been developed (Figure 1). Dasatinib, nilotinib and bosutinib provide durable salvage therapy for about half of the patients for whom imatinib fails in CML-CP, but not for those with progression to CML-BP [20,21]. Subsequent studies that compared dasatinib or nilotinib with imatinib in frontline CML-CP revealed more profound responses and reduced rates of transformation to CML-BP for the second-generation TKIs, but have yet to show differences in overall survival [22,23]. The most recent addition to the CML armamentarium is the third-generation TKI ponatinib [24]. This drug is highly active, even in patients with resistance to multiple TKIs. However, as for all other BCR-ABL1 inhibitors, although responses are durable in CML-CP, they are only transient in CML-BP [25]. In 2014, most patients diagnosed with CML-CP can expect to achieve durable responses to TKIs, and their long-term prognosis is good. A minority of patients, however, do not respond effectively to multiple TKIs or progress to CML-BP. Thus, although TKIs have improved the survival and quality of life for many CML patients, a better understanding of TKI resistance and the mechanisms leading to blastic transformation will be crucial for improving outcomes.

## Resistance to TKIs

TKI resistance in CML involves two fundamentally different mechanisms. First, BCR-ABL1 kinase-dependent resistance is driven by reactivation of BCR-ABL1 kinase activity. This typically occurs as the result of missense mutations in the kinase domain that impair drug binding through steric hindrance or conformational changes, or through BCR-ABL1 genomic amplification [26]. Other mechanisms include impaired drug influx or increased drug efflux. For example, OCT-1, a cation transporter, has been implicated in transmembrane transport of imatinib, and reduced activity or expression of this protein is associated with drug resistance [27,28]. Conversely, high expression of MDR1 is associated with nilotinib resistance [29].

**Figure 2:**
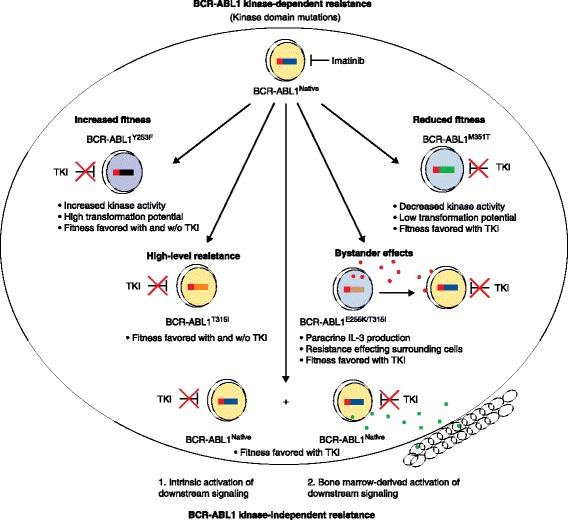
**Multiple mechanisms of tyrosine kinase inhibitor (TKI) resistance in chronic myeloid leukemia.** The schematic portrays multiple mechanisms of TKI resistance, including BCR-ABL1 kinase-dependent mechanisms (top) and BCR-ABL1 kinase-independent mechanisms (bottom). Certain tyrosine kinase mutations impart increased or decreased fitness on the BCR-ABL1 kinase. Other mutations such as T315I impart high-level resistance to first- and second-generation TKIs. Cells that carry resistance mutations may impart resistance on neighboring bystander cells by secretion of paracrine factors (such as the cytokine IL-3), so that even cells with native BCR-ABL1 become TKI resistant. Last, CML cells may acquire resistance through intrinsic activation of alternative signaling pathways or through interaction with the bone marrow microenvironment. Red and green dots denote paracrine factors produced by leukemic cells or the bone marrow microenvironment.

Second, BCR-ABL1 kinase-independent resistance is thought to occur when alternative signaling pathways are activated that maintain cell proliferation and viability despite continued suppression of BCR-ABL1 kinase activity (Figure 2) [3]. Evidence suggests that both extrinsic and intrinsic mechanisms are involved in BCR-ABL1 kinase-independent resistance and may activate the same downstream signaling molecules. Multiple extrinsic and intrinsic signals and pathways have been implicated, including JAK/STAT [30–32], phosphatidyl inositol 3′ kinase (PI3K) [33], Wnt/β-catenin [34–36], SHP-1 [37], SRC family kinases such as Lyn [38], and polymorphisms of the pro-apoptosis protein BIM [39]. The mechanistic heterogeneity of BCR-ABL1 kinase-independent TKI resistance poses a diagnostic and therapeutic challenge. Hence, individualized TKI therapy as it exists today centers on BCR-ABL1 kinase domain mutations, and BCR-ABL1 kinase-dependent resistance will be the focus of this review.

## BCR-ABL1 kinase domain mutations

### Differential TKI activity against BCR-ABL1 mutants

More than 50 different BCR-ABL1 mutations have been identified in patients with clinically manifest resistance to imatinib, but a much smaller set of mutations accounts for most acquired resistance [3]. Solving the structure of the ABL1 kinase domain crystallized with imatinib was critical to understanding mutation-based TKI resistance [40]. Unexpectedly, imatinib was found to bind an inactive conformation of ABL1, with the activation loop in a closed conformation and extensive downward displacement of the ATP-binding loop. Multiple residues are engaged by imatinib through hydrogen bonds or hydrophobic interactions, providing ample opportunity for point mutations to impair drug binding. In contrast to imatinib, which is vulnerable to a large number of different mutations, the spectrum of resistance mutations is much more limited for the second-generation TKIs, dasatinib, nilotinib and bosutinib [3]. For dasatinib, a type I inhibitor, resistance is reduced by binding to the active ABL1 conformation [41], which places less stringent requirements on inhibitor binding and hence is less liable to mutational escape. Although the conformation of ABL1 that is bound by nilotinib resembles that of the ABL1-imatinib complex, a much-improved topographic fit provides additional free energy, thereby moving many BCR-ABL1 mutants into the range of achievable nilotinib plasma concentrations [42]. Interestingly, bosutinib binds both the active and inactive conformations of ABL1 kinase [43].

**Figure 3:**
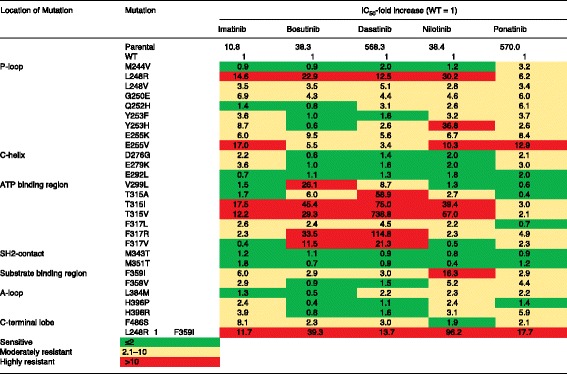
**Activities of imatinib, bosutinib, dasatinib, nilotinib, and ponatinib against mutated forms of BCR-ABL1.** Half maximal inhibitory concentration (IC_50_) values for cell proliferation of the indicated TKIs are shown against BCR-ABL1 single mutants. The color gradient demonstrates the IC_50_ sensitivity for each TKI relative to its activity against cells expressing native BCR-ABL1. Note that clinical activity is also dependent on additional factors, such as the drug concentrations achieved in the plasma of patients. Adapted with permission from Redaelli *et al*. [57].

Despite many improvements, all second-generation TKIs share a common vulnerability with imatinib, namely the T315I mutation of the ‘gatekeeper’ residue in ABL1 [3]. Substitution of threonine 315 with isoleucine prevents the formation of a key hydrogen bond (or van der Waals interaction in the case of bosutinib) between the kinase and the TKI drug, resulting in high-level resistance to multiple TKIs (Figure 2). Additionally, access to a hydrophobic pocket that is engaged by all first- and second-generation TKIs is blocked by this substitution. Ponatinib, the only third-generation TKI approved to date, is a type II inhibitor that was designed to avoid T315 by inclusion of a rigid triple carbon bond (Figure 1) [24]. Higher concentrations of ponatinib are required for inhibition of certain BCR-ABL1 mutants (for example, E255V), but these are still within the range of plasma concentrations achievable in patients, and clinical responses have been observed in patients who harbor these genotypes [24]. *In vitro* assays based on culturing cells that express randomly mutagenized BCR-ABL1 in the presence of TKIs are remarkably accurate in predicting clinically relevant BCR-ABL1 resistance mutations and contact points between TKIs and the kinase domains. Mutagenesis is achieved either by initial expression of a BCR-ABL1 plasmid in a mutagenic bacterial strain or by exposing the BCR-ABL1-expressing cells to N-nitroso-N-methylurea (ENU). Despite the fact that *in vivo* activity is dependent on multiple additional factors, including bioavailability, achievable plasma concentrations, transmembrane transport and protein binding, the *in vitro* drug sensitivity of cell lines (typically the pro-B cell line BaF/3, engineered to express BCR-ABL1 mutants in comparison to the native BCR-ABL1 kinase) is generally correlated with clinical activity (Figure 3). This allows rational TKI selection on the basis of the patient’s *BCR-ABL1* genotype, and provides an example of how molecular knowledge can aid the personalization of cancer therapy.

### Low-level BCR-ABL1 mutations

It seems logical that it would be beneficial to detect resistance mutations as early as possible, as appropriate changes can then be made to treatment strategies at an early stage to halt the expansion of a resistant clone. Given the low sensitivity of Sanger sequencing (approximately 20%), considerable effort has been dedicated to designing more sensitive assays that use a range of different technologies, including denaturing high-performance liquid chromatography (HPLC), allele-specific PCR, allele-specific ligation PCR, MassArray (Sequenom, San Diego, CA, USA) and most recently next-generation sequencing (NGS) [44–49]. These studies generally suggest that resistance mutations that are detected at low levels are predictive of less profound responses and subsequent relapse. Nevertheless, mutations detected at very low levels by allele-specific or ligation PCR were not predictive of subsequent TKI resistance [44,49]. Some of these low-level signals may be false-positive results, but an alternative explanation is that they might originate from cells that do not have full leukemogenic potential. Thus, expansion of a mutant clone to a biologically significant level may be required to validate its leukemogenic fitness. For instance, kinase domain mutations may be acquired by transiently expanding short-term leukemic stem cells (LSCs) that are unable to sustain leukemic hematopoiesis. If markers become available to select BCR-ABL1-positive LSCs, single-cell sequencing may supply critical information in this area in the future. On the other hand, low-level BCR-ABL1 kinase-domain mutations may be a marker of genetic instability, and thus the presence of multiple low-level mutations may predict a poor response to second generation TKIs [46]. Given these uncertainties, the clinical utility of high-sensitivity mutation screening is currently unclear and more prospective studies will be needed to clarify the value of this technique.

### BCR-ABL1 kinase domain mutations and clonal fitness

Several common resistance mutations localize to critical structural elements of the BCR-ABL1 enzyme, such as the ATP-binding and activation loops, and have been shown to alter the catalytic activity of the kinase. For example, certain ATP-binding loop mutations such as Y253F can increase intrinsic kinase activity to levels above that of the native kinase [50,51]. Other mutations, such as M351T, reduce intrinsic kinase activity. Results for the T315I mutation are inconsistent, probably reflecting differences in the techniques used to purify the proteins that have been subjected to enzyme-kinetic assays [50,51]. The competitiveness of cells that express BCR-ABL1 kinase mutants in a given TKI environment will reflect a balance between the gains afforded by TKI resistance with changes in kinase catalytic activity (Figure 2). Reduced kinase activity may be a critical factor that limits the acquisition of additional mutations and requires further investigation.

### Compound mutations

In Sanger sequencing traces, the presence of compound mutations (that is, two or more mutations in the same *BCR-ABL1* molecule) is inferred if the percentages of mutant alleles combined, based on their peak height relative to that of the native sequence, exceed 100%. If the combined mutant alleles are less than 100%, Sanger sequencing cannot distinguish between compound mutations and polyclonal mutations (that is, multiple BCR-ABL1 mutant clones). A widely used method to ascertain that two mutations localize to the same *BCR-ABL1* allele is shotgun cloning of *BCR-ABL1* PCR products followed by sequencing of individual colonies; however, long-range NGS may provide a less tedious approach in the future [47].

**Figure 4:**
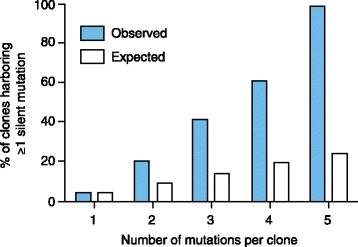
**Silent mutations increase with the total number of mutations per cell clone.** The graph represents the total number of silent mutations per clone (x-axis) and the percentage of clones with at least one silent mutation (blue bars). White bars represent the expected percentage of mutations. Adapted with permission from Khorashad *et al*. [52].

Colony sequencing has been used to demonstrate linear clonal evolution in several patients who developed multidrug-resistant compound mutant clones [52]. Interestingly, the likelihood that an additional mutation is silent rather than missense increases with the total number of mutations in the BCR-ABL1 molecule (Figure 4). This suggests that the fitness of the BCR-ABL1 kinase must ultimately be compromised by the acquisition of successive missense mutations, leading to evolutionary dead ends. From a therapeutic standpoint, this is good news as it suggests that mutational escape of the primary target kinase is not unlimited. As the impact on kinase fitness of two mutations in the same *BCR-ABL1* allele is unpredictable, experimental validation is required [53].

Compound mutations containing a T315I component confer high-level resistance to all approved TKIs, posing a considerable clinical challenge [54]. Fortunately, it seems that most compound mutations identified in patients are composed from a core set of single resistance mutations, suggesting that the number of catalytically viable combinations is limited [55]. The hope is that structural commonalities exist between subsets of possible mutations, which will allow the generation of TKIs that effectively target multiple compound mutants.

### Bystander effects of BCR-ABL1 kinase mutant clones

Some patients develop clinical resistance, although only a minority of the BCR-ABL1 amplicons found in such patients are kinase domain mutants. Two explanations for this come to mind. First, multiple resistant clones may co-exist, some with kinase domain mutations and some with BCR-ABL1 kinase-independent TKI resistance. Second, kinase domain mutant subclones may generate paracrine factors such as IL-3 that promote the survival of bystander cells. Evidence for the latter has been found *in vitro* for clones carrying the E255K/T315I compound mutation [56]. If confirmed *in vivo*, this could add another level of complexity, as resistant clones could enhance the fitness of sensitive clones by altering their microenvironment (Figure 2).

## Individualizing TKI therapy for CML

CML is one of few cancers with a close correlation between morphology and the causal genetic abnormality, which greatly facilitates the accrual of fairly homogenous patient populations for clinical studies. As imatinib, nilotinib and dasatinib are all approved for patients with newly diagnosed CML-CP, drug selection for initial therapy depends on disease risk and co-morbidities. Many attempts have been made to develop molecular prognostic markers, but the risk stratification of CML-CP patients is still largely based on clinical scoring systems such as the Sokal score, which is based on age, platelet count, spleen size and peripheral blood blast count [4]. Patients who have intermediate or high Sokal risk scores stand to benefit from second-generation TKIs, in terms of progression-free survival, whereas patients with low risk scores have excellent outcomes with all three TKIs [22]. Patients presenting with CML-BC should be treated with a second-generation TKI, typically combined with chemotherapy. Certain co-morbidities are absolute or relative contraindications for certain TKIs. For example, a prolonged heart-rate corrected QT (QTc) interval is a contraindication for the use of nilotinib, and a history of pleural effusions is a contraindication for the use of dasatinib [22].

Upon disease progression, *BCR-ABL1* genotyping is crucial for selection of the optimal TKI as salvage therapy. Recommendations are based on activity comparisons *in vitro*, typically half maximal (IC_50_) or 90% of maximal (IC_90_) inhibitory concentration values determined in BaF/3 cells expressing BCR-ABL1 mutants. Most commonly, TKI activity against a mutant is semi-quantifiable in relation to the native kinase, which permits a relative ranking of TKI activities despite different dose ranges (Figure 3) [57]. Although these assays ignore important *in vivo* factors, such as protein binding, they are indeed clinically useful. For example, V299L predicts poor response to dasatinib, E255K/V poor response to nilotinib, and T315I failure with imatinib and all second-generation TKIs, making T315I-mutant CML a prime indication for selection of ponatinib [3]. It is worth noting, however, that the correlations are tight only toward the negative side (that is, prediction of resistance). By contrast, a substantial proportion of patients with ‘sensitive mutants’ fail to respond to the respective TKI, indicating that resistance is multifactorial and presumably involves BCR-ABL1 kinase-independent mechanisms that are not measured by the currently available diagnostic assays. *Ex vivo* screening of leukemia cells using short hairpin RNAs (shRNAs) that silence kinase sequences or kinase inhibitor library panels may uncover novel therapeutic targets [58,59]. Ironically, ponatinib as a ‘pan BCR-ABL1 inhibitor’ with activity against all single mutants, including T315I, appeared to avoid the complexity of selecting the appropriate TKI for salvage; but the drug’s recently reported cardiovascular toxicity now mandates a thorough balancing of its excellent activity against the risk of potentially serious adverse events [60].

## Translating the CML paradigm to other malignancies

Kinase-targeted therapies have been approved for a range of malignancies, but few have shown activity that is comparable to that achieved in CML (Table 2). The most convincing results were seen in relatively indolent conditions, such as chronic lymphocytic leukemia (CLL) [61], hypereosinophilic syndrome [62], and myeloproliferative neoplasms with rearrangements of the platelet-derived growth factor receptors (PDGFRs) [63]. Hairy cell leukemia, which is almost universally positive for the V600E mutation in *BRAF*, may become another example, as profound responses have been reported even in chemotherapy-refractory cases [64,65]. Clinically, these conditions resemble CML in their chronic course and in the trend to progress to a more advanced stage. Biologically, the key similarity may be that constitutive activation of the target kinase is an early event in disease evolution, and is both necessary and sufficient for disease induction. Interestingly, point mutations in the target kinase BTK have been identified in CLL patients for whom ibrutinib has failed [66], and FIP1L1-PDGFα mutations in patients with hypereosinophilic syndrome for whom imatinib has failed [67]. Point mutations in FLT3 have also been reported in acute myeloid leukemia (AML) patients harboring FLT3 internal tandem duplications who relapsed after a transient response to quizartinib, a potent FLT3 inhibitor, suggesting that at least some AML patients may acquire these mutations early during disease evolution [68].

**Table 2:** **Approved indications for kinase-targeted therapies**

**Disease**	**Kinase target**	**Approved inhibitors**
Chronic myeloid leukemia (CML)	BCR-ABL1	Imatinib, dasatinib, nilotinib, bosutinib, ponatinib
Ph acute lymphocytic leukemia (ALL)	BCR-ABL1	Imatinib, dasatinib, nilotinib, bosutinib, ponatinib
Mastocytosis	KIT	Imatinib
Hypereosinophilic syndrome (HES)	FIP1L1-PDGFRα	Imatinib
Chronic eosinophilic leukemia (CEL)	FIP1L1-PDGFRα	Imatinib
	PDGFRβ	Imatinib
Gastrointestinal stromal tumors (GIST)	KIT; PDGFRα	Imatinib
Melanoma	BRAF	Vemurafenib
Non-small cell lung cancer (NSCLC)	EGFR1	Gefinitinib, erlotinib
	ALK	Crizotinib, ceritinib
Chronic lymphocytic leukemia (CLL)	BTK	Ibrutinib
Mantle cell lymphoma	BTK	Ibrutinib

BCR, breakpoint cluster region; ABL1, Abelson murine leukemia viral oncogene homolog 1; KIT, c-kit proto-oncogene; FIP1L1, FIP1-like 1; PDGFRa, platelet-derived growth factor receptor alpha; PDGFRb, platelet-derived growth factor receptor beta; BRAF, B-Raf proto-oncogene; EGFR1, epidermal growth factor receptor 1; ALK, anaplastic lymphoma kinase; BTK, Bruton’s tyrosine kinase.

At the opposite end of the spectrum of kinase-targeted therapy in hematologic malignancies is myelofibrosis. Activation of JAK/STAT signaling is universal in this disease as a result of mutations in JAK2 [69], calreticulin [70,71], or MPL [72], and JAK2 inhibitors improve clinical symptoms and possibly survival. Nevertheless, these drugs have to date failed to induce profound responses that include reduction of the malignant clone or disease burden [73,74]. Several explanations may account for the relatively disappointing results, including the genetic complexity of myelofibrosis, suppression of residual normal hematopoiesis as a result of JAK2 inhibition and the relatively low potency of available JAK2 inhibitors [75,76].

The situation is similar in solid tumors. While imatinib is active in metastatic gastrointestinal stromal tumors (GISTs), which are characterized by mutations in KIT or PDGFRα, complete responses are rare and resistance typically develops after 1 to 2 years [77]. Most melanomas with BRAF mutations are responsive to RAF inhibitors, such as vemurafenib, but complete responses are uncommon and remissions are typically transient [78]. Similarly, non-small cell lung cancers (NSCLCs) with EGFR1 mutations respond to gefitinib or erlotinib [79], and those with ALK mutations respond to crizotinib or ceritinib [80,81], but most responses are incomplete and not sustained [82]. A plethora of mechanisms have been implicated in the kinase inhibitor resistance of solid tumors. Although point mutations in the target kinase do occur (for example, in KIT and PDGFRα in GISTs [77], or EGRF and ALK in NSCLCs [83,84]), they are generally less common than alternative pathway activation, and selection of rational salvage therapies poses a greater challenge.

Why some malignancies are much more likely than others to acquire resistance by reactivation of the target kinase is unknown, but the reason is likely to be multifactorial. For example, in the case of BRAF, the specific mechanism of kinase activation promotes resistance through heterodimer formation and subsequent RAS activation [85]. Another factor may be the complexity of the signaling network operated by the activated kinase. In the case of BCR-ABL1 in CML, it may be challenging for the leukemia cells to adequately replace a large multi-domain protein with alternative signaling pathways, driving resistance toward BCR-ABL1 mutational escape [3]. In other cancers, such as AML, the presence of multiple fully oncogenic but genetically diverse clones may lead to resistance through clonal selection on therapy; alternatively, a high level of genetic instability may promote linear clonal evolution toward a drug-resistant phenotype [86]. In the future, detailed knowledge of the likely escape mechanisms for a given therapy may impact drug selection and the sequencing of active targeted agents.

## Conclusions and future directions

Current therapy of CML involves five approved TKIs that are used according to risk, disease stage, co-morbidities and *BCR-ABL1* genotype, reflecting the high level of personalization that has already been achieved in this disease. Kinase domain mutants, with their differential sensitivity to TKIs, were key drivers for this development. Clonal fitness in a given TKI environment and the intrinsic transforming capacity of the *BCR-ABL1* genotype are important determinants of drug response and resistance, providing insights into the complex interplay between drugs, malignant cells and the host that ultimately determines clinical outcomes. Current approaches to identifying resistance mechanisms to targeted cancer therapy are focused on tests that are simple to standardize for routine diagnostics, such as testing for kinase domain mutations in BCR-ABL1. Nevertheless, the detection and interpretation of low-level mutations, particularly low-level compound mutations, may be limited by the recent discovery of artifacts produced by PCR-mediated recombination events [87], a challenge that has yet to be overcome.

Characterizing drug resistance that is driven by mechanisms outside of the primary drug target is much more difficult and will predictably require genome-wide scanning technologies, such as whole-genome sequencing, or function-first assays, such as inhibitor library screens or those involving shRNAs or short interfering RNAs (siRNAs) [58]. Perhaps the greatest challenge is determining clonal complexity at diagnosis as much as at emergence of resistance. Ultra-deep sequencing and sophisticated mathematical modeling allow for reconstruction of the clonal architecture, but the resolution of this approach is ultimately limited by the error rate of the sequencing technology [88]. Overcoming this limitation will require single-cell analysis on a large scale, which is currently prohibitively expensive. In solid tumors, this is further complicated by topographic heterogeneity, which implies that multiple samples are needed to generate a representative genetic picture. Isolation and analysis of tumor cells from the blood might solve this problem in the future. Once these roadblocks have been cleared, targeted therapy will predictably move to the next level, bringing another round of fundamental change to the practice of oncology.

## Abbreviations

AML: Acute myeloid leukemia
CLL: Chronic lymphocytic leukemia
CML: Chronic myeloid leukemia
CML-BP: Blast phase CML
CML-CP: Chronic-phase CML
ENU: N-nitroso-N-methylurea
GIST: Gastrointestinal stromal tumor
HPLC: High-performance liquid chromatography
IC_50_: Half maximal inhibitory concentration
IC90: 90% of maximal inhibitor concentration
IL-3: Interleukin-3
LSC: Leukemic stem cells
NGS: Next-generation sequencing
NSCLC: Non-small cell lung cancer
PDGFR: Platelet-derived growth factor receptor
PI3K: Phosphatidyl inositol 3′ kinase
shRNA: Short hairpin RNA
siRNA: Short interfering RNA
TKI: Tyrosine kinase inhibitor
